# Violent victimization at the intersections of sexual orientation, gender identity, and race: National Crime Victimization Survey, 2017–2019

**DOI:** 10.1371/journal.pone.0281641

**Published:** 2023-02-09

**Authors:** Andrew R. Flores, Bianca D. M. Wilson, Lynn L. Langton, Ilan H. Meyer

**Affiliations:** 1 Department of Government, School of Public Affairs, American University, Washington, District of Columbia, United States of America; 2 The Williams Institute, School of Law, University of California, Los Angeles, Los Angeles, California, United States of America; 3 RTI International, Washington, District of Columbia, United States of America; Utrecht University: Universiteit Utrecht, NETHERLANDS

## Abstract

**Introduction:**

Prior research has found that experiences with violence in the U.S. differ across individual demographic characteristics, including race, gender, and sexual orientation. However, peer reviewed studies have yet to examine the relationship between the intersections of race, gender, and sexual orientation, victimization risk, and characteristics of victimization.

**Methods:**

We use data from three years (2017–2019) of the National Crime Victimization Survey, the primary source of information on criminal victimization in the United States, to examine victimization at the intersection of sexual orientation, gender, and race/ethnicity. We test whether non-Hispanic Black, Hispanic, and non-Hispanic White sexual and gender minority (SGM) persons aged 16 or over are victimized at greater rates than their non-SGM counterparts and assess whether there are differences between sexual minority females and males of each racial group. We further document characteristics of victimization such as reporting to the police by SGM status and race or ethnicity.

**Results:**

We find that SGMs are disproportionately more likely to be victims of violent crime than non-SGM people, and these disparities are present across the assessed racial and ethnic groups (non-Hispanic Black odds ratio [*OR*] = 3.3, 90% *CI* [*CI*] = 1.36, 5.16; Hispanic *OR* = 4.5, *CI* = 2.25, 6.71; non-Hispanic White *OR* = 4.8, *CI* = 2.25, 6.71). However, sexual orientation disparities are statistically distinguishable for lesbian or bisexual (LB) non-Hispanic White and Hispanic females but not for non-Hispanic Black LB females. Among LB females, the overall differences in victimization were primarily driven by bisexual respondents. We further find racial and ethnic differences among SGM victims in the likelihood of having the victimization reported to the police, in the utilization of community (non-police) resources, and in other aspects of victimization experiences, such as whether arrests occurred or in the suspicion that the violent incident was a hate crime.

**Conclusions:**

Our findings raise indicate a complex picture of how sexual orientation, gender identity, sex, and race and ethnicity interact in victimizations and their characteristics that should be further explored.

## Introduction

Prior research suggests that experiences with violence differ across gender, race, and ethnicity in the United States. Women tend to have higher rates of victimization than men; Black or African American persons historically have higher risk of victimization than non-Hispanic White people [1]. For decades, advocates and researchers have indicated that sexual orientation and gender identity are demographic characteristics that need to be added to the typical slate of social statuses examined in violence and other literatures [2, 3]. As these characteristics have been added to large federal surveys, like the National Crime Victimization Survey, data have demonstrated that sexual and gender minorities (SGM, i.e., lesbian, gay, bisexual, and transgender people) experience higher rates of victimization than their heterosexual and cisgender peers [4–6].

Across studies of victimization disparities, findings show that minority or subjugated groups (e.g., ethnic and racial minorities, cisgender women, transgender people, and sexual minorities) tend to experience victimization at higher rates than dominant social groups, such as White, cisgender men, and heterosexual people [1, 2, 4–6]. Population-based data have shown that victimization disparities between cisgender men and women and among racial and ethnic groups have declined in recent years [2]. studies have also shown disparate impact of violent victimization on physical and mental health for marginalized groups [7].

With some exceptions [8], most research on SGM populations examining victimization rates and experiences have so far been limited in assessing race/ethnicity as a factor that may affect outcomes, which may be due in part to data limitations. A remaining question from population-based data is whether differences in experiences with victimization between SGM and non-SGM groups persist at the intersection of race/ethnicity and gender, such as how SGM Hispanic people compare to non-SGM Hispanic people. Qualitative and community-based studies on the experiences with violence among transgender women indicate that transgender women of color report being targeted and victimized in ways that White transgender women, are not [9, 10]. Through 47 in-depth interviews, Meyer [11] shows the deep and interlocking systems of sexism, heterosexism, genderism, and classism that uniquely shape the victimization experience of LGBT people of color and indicates that rates and characteristics of victimization may look different across racial and ethnic groups.

Theoretical work on the impact of belonging to multiple marginalized social statuses, particularly among LGBT youth of color, indicate that being in two or more stigmatized social groups may produce compounded threats to victimization [12]; however, empirical work on this topic has shown mixed findings [13–15]. To date, no population-based peer reviewed study has assessed whether patterns of violence vary at the intersection of race/ethnicity, gender, and SGM statuses among adults. Here, we address this question using data representative of the United States population.

## Materials and methods

The National Crime Victimization Survey (NCVS) is a stratified, multi-stage cluster sample of households in the United States that surveys individuals aged 12 years and older. The US Census Bureau administers the NCVS for the Bureau of Justice Statistics. The purpose of the NCVS is to document the prevalence and characteristics of violent and property crimes in the U.S., regardless of whether such experiences were reported to the police. Data collection for the NCVS is performed on a continuous basis, with households probabilistically selected, recruited and empaneled for 3.5 years and interviewed at 6-month intervals. The sampling frame includes group quarters, such as dormitories, but excludes military base housing and institutional settings (e.g., correctional or medical facilities). Households are sampled and recruited monthly, with one-seventh of the empaneled households rolling off and being replaced by a new sample of households.

Once a household is sampled, all eligible respondents 12-year-old or older are interviewed. From 2017 to 2019 NCVS respondents aged 16-year-old or older were asked three questions relating to their sexual and gender identities. Thus, the sample for the current study includes individuals 16-year-old or older.

The US Census Bureau field representatives conduct the interviews either in person or over the telephone. Usually, the first survey is conducted in person while the follow-up surveys are conducted via telephone. The 2017 NCVS contains 145,508 household interviews and 239,541 individual interviews, representing 76% of eligible households and an individual response rate 84% [16]. The 2018 NCVS contains 151,055 household interviews and 242,928 individual interviews, representing 73% of eligible households and an individual response rate of 82% [17]. The 2019 NCVS contains 151,076 households interviews and 249,000 individuals interviews, representing 72% of eligible households and an individual response rate of 83% [18]. The Bureau of Justice Statistics reports on criminal victimization [19] and their technical documentation [20] further detail the design and implementation of the NCVS.

The NCVS public-use files are available for download in the National Archive of Criminal Justice Data hosted by the Inter-University Consortium for Political and Social Research at the University of Michigan. Since the current analyses are a secondary analysis of publicly available survey data, the study is not considered human subjects research for ethical review purposes and is exempt from human subjects’ review, and the researchers did not engage in primary data collection and thus did not obtain consent.

### Measures

#### Sexual orientation and gender identity

Sexual orientation can be measured in surveys by asking about people’s sexual identities, behaviors, and/or attractions. In the NCVS, sexual identity was measured by asking, “Which of the following best represents how you think of yourself?” with the following response options, “lesbian or gay,” “straight, that is, not lesbian or gay,” “bisexual,” “something else,” or “I don’t know the answer” (variable name: V3084). Of men in the pooled sample, 1.6% (*n* = 3,737) were gay, 0.3% (*n* = 799) were bisexual, 95.8% (*n* = 247,098) were straight, and 2.3% (*n* = 5,360) responded as something else, do not know, or refused to answer this question. Of women in the pooled sample, 1.1% (*n* = 3,031) were lesbian, 1.0% (*n* = 2,462) were bisexual, 95.5% (*n* = 279,832) were straight, and 2.5% (*n* = 6,713) responded as something else, do not know, or refused to answer this question.

Gender identity was measured via a two-step procedure, where respondents are asked their sex assigned at birth and then asked their current gender identity [21]. In the NCVS, sex assigned at birth was measured by asking, “What sex were you assigned at birth, on your original birth certificate?” with response options, “male,” “female,” and “don’t know.” Current gender identity was measured by asking, “Do you currently describe yourself as male, female, or transgender?” with response options, “male,” “female,” “transgender,” or “none of these.” Respondents are categorized as transgender if they indicated that they were transgender or that they have a current gender identity that was different from their sex assigned at birth. Of the pooled sample, 0.10% (*n* = 535) were categorized as transgender and 99.9% (*n* = 553,390) as cisgender.

Sexual and gender identity measures were included in the survey after pre-implementation cognitive checks and during implementation checks such as focus groups, interviewer trainings, interviews with interviewers, and cognitive testing, which evaluated how the inclusion of these measures would affect overall data quality of the NCVS [22, 23].

For the aims of this study, we categorized respondents as a SGM if they either identified as lesbian, gay, or bisexual or if they were categorized as transgender. We categorized respondents as non-SGM if they identified as straight and were classified as cisgender. Respondents who identified as “something else” in the sexual orientation question or “none of these” in the gender identity question but otherwise were not an SGM, were categorized separately from non-SGMs and SGMs as “Unknown/Other.” We report results for this group in S1 Appendix. In the pooled sample, 2.1% (*n* = 10,533) were SGMs, 94.8% (*n* = 527,846) were non-SGMs, and 3.1% (*n* = 15,546) were Unknown/Other.

#### Race and ethnicity

The NCVS measures the race of respondents with the following question and response options, “Please choose one or more races that you consider yourself to be: “White,” “Black or African American,” “American Indian or Alaska Native,” “Asian,”, or “Native Hawaiian or Other Pacific Islander.” The person-level file contains a summary measure of race including whether and what multiple categories of race were selected (variable name: V3023A).

Respondents are separately asked, “Are you Spanish, Hispanic, or Latino?” with response options, “yes” or “no” (variable name: V3024). For this analysis race and ethnicity were combined into a summary measure to categorize respondents as non-Hispanic, monoracial White (63.5%, *n* = 388,359), non-Hispanic Black inclusive of multiracial or biracial persons (12.5%, *n* = 61,961), Hispanic (16.3%, *n* = 69,397), and other race or multiracial (7.7%, *n* = 34,208). For sample size purposes, we restrict our analyses to non-Hispanic White, non-Hispanic Black, and Hispanic respondents. Our decision to categorize multiracial or biracial non-Hispanic Black respondents as Black is to create an inclusive category reflecting an experience of potentially being perceived as Black. Research shows that the experience of blackness and anti-black prejudice are, in part, a function of other people’s perceptions of one’s race and that many black multiracial people describe experiences of social treatment aligned with monoracial black people’s reports [24, 25].

#### Violent victimization

Once it has been determined that a person was a victim of a crime in the screening questionnaire, the incident questionnaire is used to gather greater details about the crime and to classify it. The Bureau of Justice Statistics maintains standard classifications of crime [20], which are used in its annual reports on criminal victimizations [19]. We follow these standard classifications to categorize types of crime, which involves recoding a type of crime variable in the incident-level file (variable name: V4529). There are broadly two types of criminal victimization: violent victimizations and property victimizations (there is also personal larceny, which is not addressed in our analyses). Violent victimizations comprise serious violent victimizations and simple assaults. A simple assault is an attack, attempted attack, or verbal threat to attack a victim that does not involve a weapon and that results in minor injury with less than two days of hospitalization. Serious violence consists of crimes of rape or sexual assault, robbery, aggravated assault, victimizations involving a weapon, and victimizations that result in injury requiring three or more days of hospitalization. For sample size purposes, we restrict our analyses to broader categorizations of violent victimizations including all violent victimizations, simple assaults, serious violence, and serious violent victimizations that involve injuries.

#### Victim-offender relationship

In the incident questionnaire, victims are asked about the relationship they had with the perpetrator or perpetrators. Respondents are asked to specify one of six categories to indicate whether and how well the victim knew the offender. The six categories include a stranger, a person whom the victim has seen before but has had little-to-no interaction, a casual acquaintance, well-known non-relative (i.e., closer than an acquaintance), a relative, and an intimate or former intimate partner. In cases of multiple perpetrators, victims are asked about the nature of the relationship with each of the offenders. If at least one offender is well-known, then the incident is considered to be one where the victim-offender relationship is well-known. A well-known victim-offender relationship is also subdivided into the following categories: intimate partners (i.e., spouses, ex-spouses, boyfriends/girlfriends, or former boyfriends/girlfriends), relatives, or other well-known offenders. To address small sample sizes, we combine victim-offender relationship and describe two categories: offenders who are strangers or are well-known to the victim.

#### Police involvement and other victimization characteristics

In addition to obtaining details about a victimization for appropriate crime classification, the NCVS gathers greater details about the victimization. In this study, we examine whether the victimization was reported to the police, why it was reported or not, whether it was categorized as a hate crime, whether an arrest was made, and whether the respondent sought help or support for crime-related trauma.

#### Other demographic characteristics

We considered the following demographic characteristics. **Respondent sex** was measured dichotomously with 51.9% (*n* = 294,638) being female and 48.1% (*n* = 259,287) being male (variable name: V3018), which was collected on the control card and was a different measure than assigned sex at birth. **Age cohort** was measured from a continuous measure of the respondent’s age (variable name: V3014) discretized to age groups with 3.0% (*n* = 10,021) aged 16–17 years old, 11.2% (*n* = 41,758) aged 18–24 years old, 17.4% (*n* = 83,458) aged 25–34 years old, 24.1% (*n* = 132,699) aged 35–49 years old, 24.7% (*n* = 149,696) aged 50–64 years old, and 19.6% (n = 136,293) aged 65 years old or more. **Educational attainment** was measured as the respondent’s highest year of completed education (variable name: V3020) recoded into the following groups: less than a high school diploma (14.9%, *n* = 71,571), high school graduate (25.1%, *n* = 138,691), some college, a two-year degree, or a vocational degree or certificate (27.8%, *n* = 153,051), a bachelor’s degree (20.8%, *n* = 115,843), and a graduate or professional degree (11.5%, *n* = 68,163). **Marital status** was measured as the current relationship status of the respondent (variable name: V3015) with 50.0% (*n* = 302,279) married, 30.5% (*n* = 132,377) never married, 5.8% (*n* = 39,170) widowed, 10.9% (*n* = 66,079) divorced, and 2.0% (*n* = 10,534) separated. **Household income** (variable name: SC214A) and **urbanicity** (variable name: V2129) were measured at the household level. Following BJS practices, these variables were merged to the person-level file and applied to each individual respondent. The income distribution was rescaled to the eight following income categories, coded 1 = “less than $9,999,” 2 = “$10,000-$14,999,” 3 = “$15,000-$24,999,” 4 = “$25,000-$34,999,” 5 = “$35,000-$49,999,” 6 = “$50,000-$74,999,” 7 = “$75,000-$99,999,” and 8 = “$100,000 or more.” The median income category was $50,000-$74,999 at both the household level and person level. Urbanicity was measured as a three-level variable with 33.6% (*n* = 168,321) in a city of a metropolitan statistical area (i.e., urban), 52.3% (*n* = 305,523) in a metropolitan statistical area but not in a city (i.e., suburban), and 14.1% (*n* = 80,081) not in a metropolitan statistical area (i.e., rural).

#### Data weighting and analysis

The Census Bureau applies weights to the data file, which account for the complex design of the NCVS and adjust for non-response bias and unequal probabilities of selection. The weights are also used to create population estimates of households (i.e., household weights), persons (i.e., person weights), criminal incidents (i.e., incident weights), and victimizations (i.e., victimization weights). We apply these weights, as appropriate, for all estimates. Standard errors are estimated via direct variance estimation via Taylor series linearization, taking into account the complex design of the survey and following the steps outlined in a technical report for direct variance estimation [26].

We provide summary statistics of demographic characteristics for SGMs and non-SGMs and by race or ethnicity. We indicate significant differences in proportions from Student’s *t*-tests, and we also report tests of independence as a Rao-Scott *F-*test [27]. We report victimization rates, which is the number of victimizations per 1,000 persons. We report differences in the victimization rates between SGM and non-SGMs. We report an odds ratio (*OR*) comparing SGM to non-SGM estimated victimization rate $\left(\hat{VR}\right)$ with the following calculation [5]:

$$\begin{array}{r} OR=\frac{{\hat{VR}}_{SGM}}{1000-{\hat{VR}}_{SGM}}÷\frac{{\hat{VR}}_{Non-SGM}}{1000-{\hat{VR}}_{Non-SGM}}. \end{array}$$

We also report rates of violent victimization by sexual orientation (LGB vs. straight) and sex (male or female). For sample size purposes, we do not further disaggregate by gender identity for those analyses. Throughout, we report 90% *CI* (*CI*), and we deem differences as significant with a one-tailed *p* < .05. We select this level of significance because prior examinations of the NCVS consistently report victimization disparities between SGMs and non-SGMs [4–6].

Characteristics of victimizations examine what proportion of violent victimizations had a certain characteristic, and we indicate significant differences in proportions from Student’s *t*-tests and with *OR*s from logistic regressions. We document in S2 Appendix regression results on victimization rates and adjusted victimization rates controlling for respondent demographic characteristics. Since only respondents aged 16 years of age or older were asked about their sexual orientation and gender identity, we only report results for individuals aged 16 years or older throughout this article. We follow BJS standards for flagging estimates that are unreliable [20]. Estimates are considered unreliable if they are based on 10 or fewer observations or if the size of the standard error relative to the point estimate is greater than or equal to 50%.

We perform our analyses subdividing to the three largest racial and ethnic groups in the United States: White, non-Hispanic populations, mono-, bi-, or multi-racial Black, non-Hispanic populations, and Hispanic or Latino populations. We further analyze sexual orientation differences by sex.

In 2019, BJS changed the approach for how respondents were asked about their sexual orientation and gender identity. Beginning in July 2019, sexual orientation and gender identity were asked only of those who responded affirmatively to a crime screener questions, suggesting that they were a victim of crime. Those who were not identified as victims were not asked the questions, meaning that the NCVS could no longer be used to generate a population denominator for SGM victimization rates after July 2019. To account for this change, we used data collected from January 2017 through June 2019 to estimate demographics and victimization rates. All other estimates use the entire timeframe.

Our analyses were conducted in STATA SE v.14.2, and we relied on the *svy*: command for direct variance estimation. We further relied on the *subpop* option to analyze observations that met our inclusion criteria (e.g., 16 years of age or older, within a specified time frame, interviews were not proxy interviews, and victimizations that occurred within the United States).

## Results

### Demographic characteristics

The demographic breakdown of the sample subdivided by SGM status for non-Hispanic White, non-Hispanic Black, and Hispanic people are in Table 1. White SGMs tend to be younger, unmarried, and residents of urban locations compared to White non-SGMs. There are some gender differences in SGM status in the Black population: Black female SGMs comprise a larger share of the SGM subgroup than Black male SGMs; Black SGMs are also more likely to be younger, unmarried, and reside in urban locations than Black non-SGMs; differences in educational attainment between Black SGM and non-SGM are not substantively large. The patterns and characteristics of the Hispanic SGM population are similar to overall SGM characteristics. Hispanic SGMs tend to be younger, at least high school graduates, unmarried, and residents of urban locations.

**Table 1:** Demographic characteristics SGMs and non-SMGs by race and ethnicity.

	Non-Hispanic White				Non-Hispanic Black				Hispanic			
	SGM		Non-SGM		SGM		Non-SGM		SGM		Non-SGM	
	**%**	*SE*	%	*SE*	**%**	*SE*	**%**	*SE*	**%**	*SE*	**%**	*SE*
**Sex**												
Male	**46.4**	0.99	**48.5** ^**a**^	0.13	**37.8**	0.25	**45.3** ^**a**^	0.41	**51.2**	2.41	**49.4**	0.37
Female	**53.6**	0.99	**51.5** ^**a**^	0.13	**62.2**	0.25	**54.7** ^**a**^	0.41	**48.8**	2.41	**50.6**	0.37
*N*	7,664		369,804		1,022		59,048		1,311		66,466	
*F*	5.36^b^				5.72^b^				0.46			
**Age**												
16–17	**4.1**	0.43	**2.4** ^**a**^	0.06	**3.1**	0.82	**3.8**	0.21	**6.7**	1.1	**4.5** ^**a**^	0.13
18–24	**19.3**	0.87	**9.2** ^**a**^	0.22	**23.2**	2.15	**13.2** ^**a**^	0.34	**27.0**	2.42	**15.2** ^**a**^	0.2
25–34	**22.7**	0.83	**15.2** ^**a**^	0.16	**35.7**	2.68	**19.3** ^**a**^	0.38	**29.1**	1.83	**21.6** ^**a**^	0.39
35–49	**21.9**	0.84	**22.3**	0.16	**22.6**	2.07	**24.6**	0.34	**20.2**	1.88	**29.4** ^**a**^	0.31
50–64	**22.6**	0.95	**26.9** ^**a**^	0.20	**12.9**	2.07	**24.1** ^**a**^	0.36	**14.4**	1.60	**19.3** ^**a**^	0.32
65 or older	**9.3**	0.60	**24.0** ^**a**^	0.22	**2.6**	0.65	**14.9** ^**a**^	0.31	**2.6**	0.65	**10.0** ^**a**^	0.29
*N*	7,664		369,804		1,022		59,048		1,311		66,466	
*F*	56.23^b^				16.73^b^				12.15^b^			
**Education**												
Less than High School	**9.9**	0.74	**10.5**	0.21	**12.7**	1.54	**17.7** ^**a**^	0.44	**19.1**	1.78	**31.2** ^**a**^	0.46
High School Graduate	**17.3**	0.84	**24.8** ^**a**^	0.32	**25.2**	2.23	**29.2** ^**a**^	0.43	**22.9**	1.65	**27.8** ^**a**^	0.38
Some College	**30.0**	0.96	**28.5**	0.21	**35.8**	2.25	**30.8** ^**a**^	0.42	**35.6**	1.79	**25.0** ^**a**^	0.40
Bachelor’s Degree	**26.4**	0.99	**23.4** ^**a**^	0.26	**19.1**	2.13	**15.0** ^**a**^	0.38	**15.6**	1.77	**11.0** ^**a**^	0.28
Post-Graduate	**16.5**	0.89	**12.8** ^**a**^	0.17	**7.2**	1.36	**7.3**	0.21	**6.7**	1.31	**5.1**	0.16
*N*	7,636		366,923		1,010		58,297		1,303		65,679	
*F*	10.13^b^				2.47^b^				8.36^b^			
**Marital Status**												
Never married	**59.9**	1.26	**24.6** ^**a**^	0.26	**77.9**	1.90	**46.2** ^**a**^	0.51	**71.3**	2.28	**35.9** ^**a**^	0.41
Married	**28.0**	1.34	**55.0** ^**a**^	0.22	**11.0**	1.62	**32.0** ^**a**^	0.45	**19.7**	1.77	**49.7** ^**a**^	0.46
Widowed	**1.3**	0.17	**6.9** ^**a**^	0.09	**0.7**	0.29	**6.1** ^**a**^	0.22	**0.5**	0.24	**3.2** ^**a**^	0.15
Divorced	**9.4**	0.65	**12.1** ^**a**^	0.12	**8.3**	1.48	**12.0** ^**a**^	0.26	**6.4**	1.06	**7.8**	0.22
Separated	**1.3**	0.19	**1.4**	0.04	**2.1**	0.60	**3.7** ^**a**^	0.14	**2.1**	0.57	**3.4** ^**a**^	0.13
*N*	7,652		368,533		1,014		58,476		1,306		66,210	
*F*	221.73^b^				27.90^b^				51.07^b^			
**Household income**												
Less than $10,000	**7.6**	0.55	**4.3** ^**a**^	0.17	**14.3**	1.34	**9.9** ^**a**^	0.37	**8.5**	1.13	**5.8** ^**a**^	0.24
$10,000-$14,999	**4.2**	0.39	**3.7**	0.87	**7.9**	1.28	**7.4**	0.25	**6.6**	1.12	**5.3**	0.17
$15,000-$24,999	**7.9**	0.54	**7.6**	0.16	**9.9**	1.20	**12.4** ^**a**^	0.30	**10.9**	1.37	**12.6**	0.37
$25,000-$34,999	**8.4**	0.51	**9.0**	0.15	**13.0**	1.39	**13.4**	0.29	**12.1**	1.46	**14.5** ^**a**^	0.31
$35,000-$49,999	**13.7**	0.70	**14.4**	0.18	**14.2**	1.56	**17.3** ^**a**^	0.39	**16.6**	1.60	**18.5**	0.35
$50,000-$74,999	**17.3**	0.75	**19.0** ^**a**^	0.17	**16.7**	1.78	**16.3**	0.35	**16.6**	1.44	**18.5**	0.41
$75,000-$99,999	**14.3**	0.74	**16.3** ^**a**^	0.19	**9.6**	1.59	**11.5**	0.32	**13.4**	1.44	**12.1**	0.32
$100,000 or more	**26.5**	0.90	**25.6**	0.32	**14.4**	1.97	**11.9**	0.38	**15.5**	1.71	**12.7** ^**a**^	0.31
*N*	7,664		369,804		1,022		59,048		1,311		66,466	
*F*	12.51^b^				1.69				2.39^b^			
**Urbanicity of Residence**												
Urban	**42.5**	1.41	**25.9** ^**a**^	0.81	**59.9**	2.80	**48.9** ^**a**^	1.62	**55.4**	2.52	**44.0** ^**a**^	0.96
Suburban	**46.1**	1.40	**55.3** ^**a**^	1.15	**35.8**	2.66	**42.5** ^**a**^	1.50	**41.3**	2.54	**50.8** ^**a**^	0.75
Rural	**11.4**	1.38	**18.8** ^**a**^	1.70	**4.3**	1.11	**8.6** ^**a**^	1.8	**3.3**	0.97	**5.2** ^**a**^	0.95
*N*	7,664		369,804		1,022		59,048		1,311		66,466	
*F*	21.98^b^				9.29^b^				7.34^b^			

Summary statistics are of the pooled January 2017- June 2019 NCVS sample aged 16 years or older.

^a^ Difference between SGM and non-SGM is statistically significant at *p* < .05 (one-tailed).

^b^ Test of independence *F*-statistic is statistically significant at *p* < .05.

### Victimization by race and ethnicity

**Fig 1:**
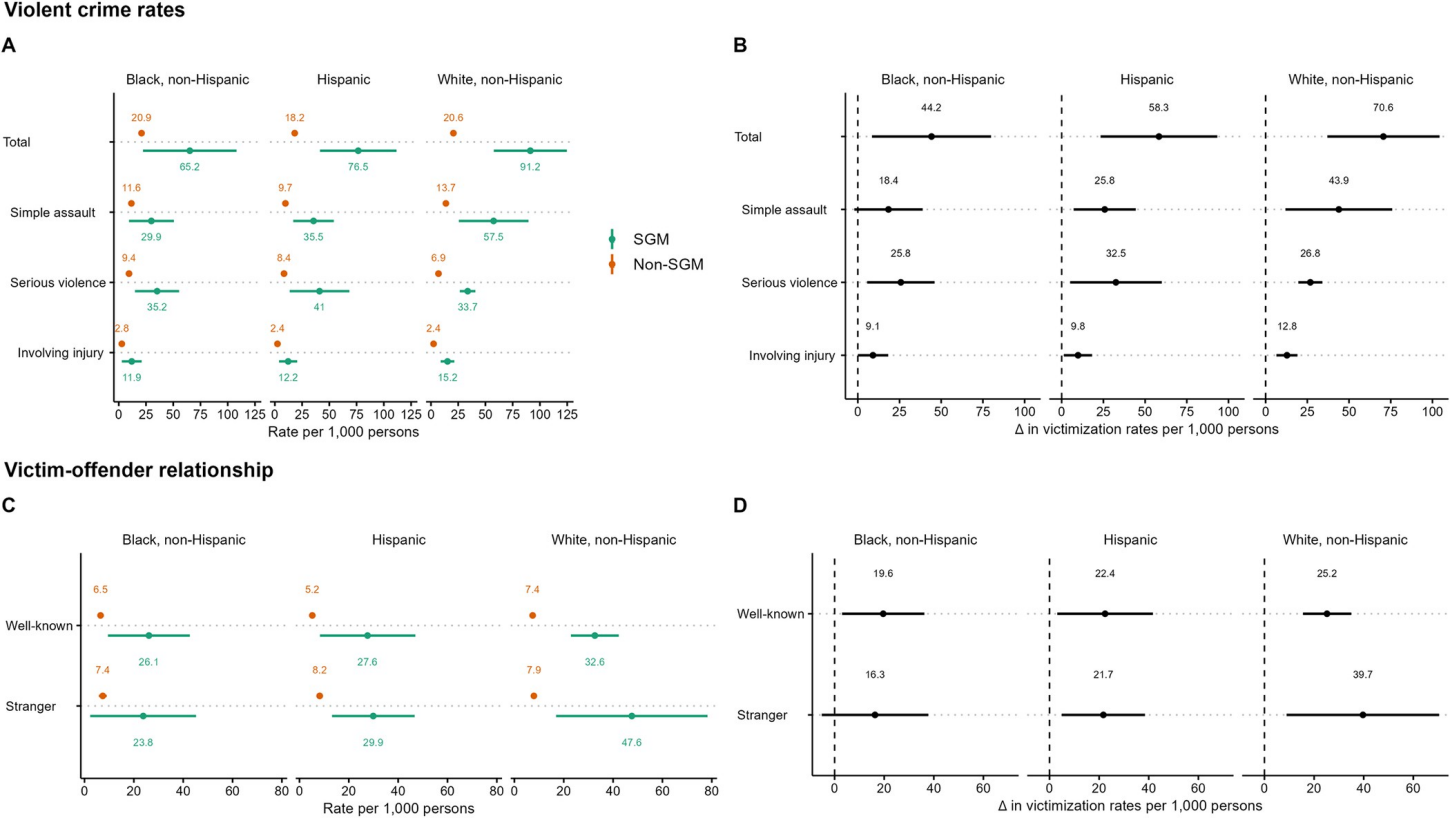
Violent victimization rates by race and ethnicity. (A) Types of violent victimization rates by SGM status and race and ethnic groups. (B) Differences in types of violent victimization rates between SGMs and non-SGMs by race and ethnic groups. (C) Victim-offender relationship victimization rates by SGM status and race and ethnic groups. (D) Differences in victim-offender victimization rates between SGMs and non-SGMs by race and ethnic groups. 90% confidence intervals represented by line segments.

We report victimization rates for select types of crime by race or ethnicity (Fig 1A) and differences in these victimization rates between SGMs and non-SGMs by race or ethnicity (Fig 1B). Overall, SGMs have higher victimization rates than non-SGMs within each racial and ethnic group in violent victimizations (Black *OR* = 3.3, 90% *CI* [*CI*] = 1.34, 5.16; Hispanic *OR* = 4.5, *CI* = 2.25, 6.71; White *OR* = 4.8, *CI* = 2.80, 6.76). However, the patterns of SGM differences in victimization are not the same in each racialized group for each subtype of violent victimization (simple assault, serious violence, and serious violence involving injury). Among Hispanic and White respondents, SGMs had higher victimization rates than non-SGMs in simple assaults (Hispanic *OR* = 3.7, *CI* = 1.66, 5.84; White *OR* = 4.4, *CI* = 1.78, 7.02), but these rates were not significant between Black SGMs and non-SGMs (*OR* = 2.64, *CI* = 0.78, 4.50). With regard to serious violent victimizations, SGMs have higher rates than non-SGMs within each racialized group (Black *OR* = 3.8, *CI* = 1.48, 6.21; Hispanic *OR* = 5.0, *CI* = 1.42, 8.63; White *OR* = 5.0, *CI* = 3.78, 6.28) and serious victimizations involving injury (Black *OR* = 3.78, *CI* = 0.77, 6.79; Hispanic *OR* = 4.2, *CI* = 0.99, 7.35; White *OR* = 6.8, *CI* = 3.88, 9.76). However, the serious violence involving injury victimization rate is based on fewer than the observations for Black SGMs, and should not be considered a reliable estimate.

Victimization rates by victim-offender relationship are also reported (Fig 1C) and differences in these victimization rates between SGMs and non-SGMs by race or ethnicity (Fig 1D). Within each racial and ethnic group, SGMs have higher rates of violent victimizations than non-SGMs involving well-known perpetrators (Black *OR* = 4.1, *CI* = 1.35, 6.85; Hispanic *OR* = 5.4, *CI* = 1.44, 9.41; White *OR* = 4.5, *CI* = 3.03, 6.06). Hispanic and White SGMs had higher rates of violent victimizations than non-SGMs involving strangers (Hispanic *OR* = 3.7, *CI* = 1.51, 5.96; White *OR* = 6.3, *CI* = 1.95, 10.58), but this was not significant between Black SGMs and non-SGMs (*OR* = 3.2, *CI* = 0.19, 6.31). We find that among White and Hispanic populations, significant disparities exist between SGMs and non-SGMs in well-known and stranger violent victimization rates. We also find among Black populations there are significant disparities between SGMs and non-SGMs in victimization rates when the perpetrator is well-known to the victim.

**Fig 2:**
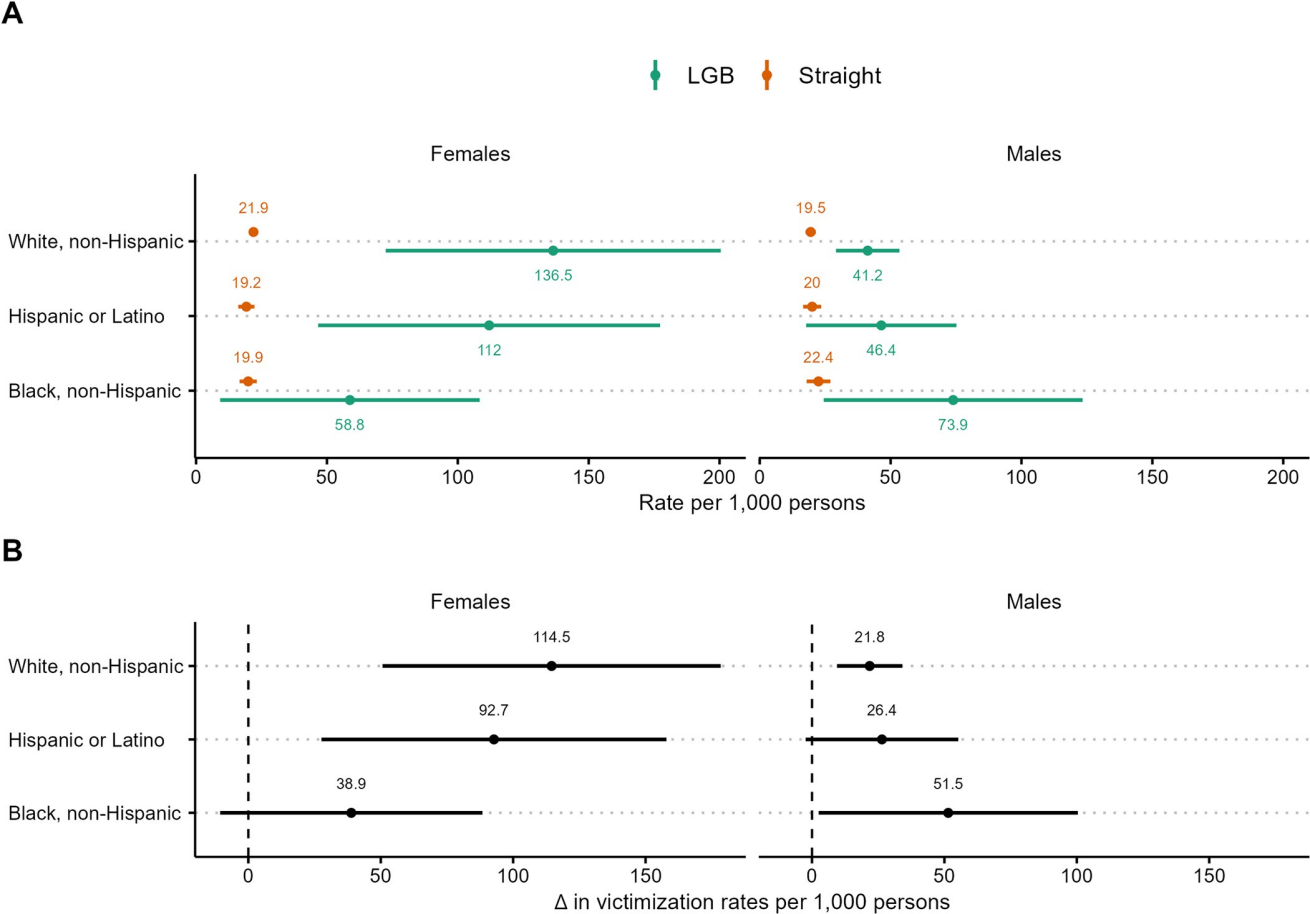
Violent victimization rates by race, ethnicity and sex. (A) Total violent victimization rates by SGM status, race, ethnicity and sex. (B) Differences in total violent victimization rates between SGMs and non-SGMs by race, ethnicity, and sex. 90% confidence intervals represented by line segments.

We report violent victimization rates by LGB status, race or ethnicity, and sex (Fig 2A) and differences in rates of violent victimization between LGB people and straight people by race or ethnicity and gender (Fig 2B). We generally find that violent victimization rates are higher for LGB people than straight people within each racial or ethnic group by sex. Among males, Black GB men have higher violent victimization rates than White, Hispanic LGB men or straight men, though differences are not statistically significant. Significant disparities between LGB and straight people are present among White males (*OR* = 2.2, *CI* = 1.46, 2.87) but not for Black males (*OR* = 3.5, *CI* = 0.99, 5.96) and Hispanic males (*OR* = 2.4, *CI* = 0.80, 3.96). However, the difference in victimization rates between LGB and straight males among White and Black men were significant (Fig 2B).

Among females, White and Hispanic LB females have higher violent victimization rates than Black LB females. Significant disparities between LB females and straight females are present among White females (*OR* = 7.1, *CI* = 3.25, 10.85) and Hispanic females (*OR* = 6.4, *CI* = 2.18, 10.69) but not for Black females (*OR* = 3.1, *CI* = 0.31, 5.84).

Among LB females, the overall differences in victimization rates were primarily driven by bisexual respondents. The rates of violent victimization for bisexual females were 133.3 (*CI* = 15.2, 251.5), 175.8 (*CI* = 58.1, 293.5), 201.1 (*CI* = 84.6, 317.7) victimizations per 1,000 people for Black, Hispanic, and White bisexual females, respectively. This resulted in significant disparities between bisexual females and straight females for Hispanic females (*OR* = 11.6, *CI* = 2.19, 21.04) and White females (*OR* = 11.3, *CI* = 3.10, 19.49) but not for Black females (*OR* = 7.70, *CI* = 0.00, 15.60). Due to sample size limitations, analyses could not be performed for other LGB subgroups (i.e., lesbians, gay men, or bisexual men) by sex and race/ethnicity.

### Police involvement and other characteristics of victimization experiences

We tested for differences based on SGM status on characteristics of violent victimization reports within each racial group (Table 2). There were very few differences between SGM and non-SGM respondents on these characteristics. However, Black SGM respondents were significantly less likely than Black non-SGM people to report an incident to police (*OR* = 0.28, *CI* = 0.12, 0.63), but they were more likely to access professional help for feelings associated with the crime (*OR* = 6.96, *CI* = 3.13, 15.47). When an incident was reported to police, Black (*OR* = 0.23, *CI* = 0.04, 1.39), Hispanic (*OR* = 0.33, *CI* = 0.10, 1.07), and White (*OR* = 0.35, *CI* = 0.19, 0.66) SGM respondents were less likely to indicate that the perpetrator was arrested than their non-SGM peers. Only White SGM respondents were more likely to suspect the incident was a hate crime compared to their non-SGM counterparts (*OR* = 4.15, *CI* = 2.27, 7.58).

**Table 2:** Victimization characteristics by SGM status and race or ethnicity.

	Non-Hispanic White				Non-Hispanic Black				Hispanic			
	SGM		Non-SGM		SGM		Non-SGM		SGM		Non-SGM	
	%	*SE*	%	*SE*	%	*SE*	%	*SE*	%	*SE*	%	*SE*
**Reported to Police**												
Yes	**47.2**	9.18	**44.1**	1.71	**24.3**	8.72	**53.6** ^**a**^	3.39	**36.0**	10.4	**48.7**	3.11
**Most Important Reason to Report (among victimizations reported to the police)**												
Get help with this incident	**17.3**	7.18	**24.8**	2.06	**15.0**	10.2	**25.7**	3.28	**11.4**	6.85	**20.9**	3.46
To get offender	**28.5**	10.0	**18.6**	1.71	**39.8**	16.66	**14.5**	2.50	**41.8**	15.25	**21.4**	3.65
Let police know	**2.6**	1.58	**4.7**	1.47	**0** ^**b**^	--	**2.4**	0.84	**0** ^**b**^	--	**3.1**	2.22
Other	**23.2**	13.52	**18.0**	1.46	**22.6**	13.79	**20.3**	4.16	**10.8**	6.59	**18.2**	2.67
**Most Important Reason to Not Report (among victimizations not reported to the police)**												
Dealt with another way	**36.6**	5.78	**35.4**	2.35	**21.9**	7.75	**30.7**	4.10	**19.5**	9.0	**26.2**	3.68
Not important enough	**27.4**	7.58	**16.7**	1.43	**17.6**	6.66	**19.4**	3.83	**10.3**	8.01	**19.5**	3.34
Police wouldn’t or couldn’t do anything	**11.6**	4.62	**16.0**	1.49	**0** ^**b**^	-	**23.7** ^**a**^	4.1	**22.2**	12.0	**22.2**	3.94
Fear of reprisal or getting offender in trouble	**15.1**	6.28	**12.7**	1.26	**23.8**	14.6	**9.4**	1.67	**16.3**	10.6	**12.6**	2.58
Other	**8.8**	3.51	**13.8**	1.79	**35.2**	12.1	**15.2**	3.3	**31.7** ^**b**^	20.2	**14.3**	2.77
**Arrest Made (among victimizations reported to the police)**												
Yes	**13.8**	4.42	**31.4** ^**a**^	2.14	**6.8**	6.72	**23.9** ^**a**^	3.84	**12.0**	7.29	**28.9** ^**a**^	3.21
**Suspect Victimization Was a Hate Crime**												
Yes	**22.4**	6.25	**6.5** ^**a**^	0.74	**14.2**	5.25	**12.6**	2.7	**25.2**	11.3	**8.0**	2.18
**Seek Professional Help for Feelings Experienced as Victim of Crime**												
Yes	**17.9**	5.03	**11.4**	1.07	**43.9**	11.16	**10.1** ^**a**^	1.72	**24.8**	10.77	**9.1**	1.69

Victimization characteristics are of victims of violent crime in the pooled January 2017- December 2019 NCVS sample aged 16 years or older.

^a^ Difference between SGM and non-SGM is statistically significant at *p* < .05 (one-tailed).

^b^ Estimate is unreliable due to insufficient observations or a large relative standard error (i.e., *RSE*≥50%).

## Discussion

This study is among the first to use population-based data to examine criminal victimization—including simple violent victimizations, serious violent victimizations, and serious violent victimizations that involve injuries—among sexual and gender minorities at the intersection of race/ethnicity and sex. This study replicates prior research showing that SGMs have higher victimization rates than non-SGMs and extends prior work [4–6] by demonstrating that these SGM disparities vary across non-Hispanic Black, Hispanic and non-Hispanic White groups [see also 8, 28]. We find that in White and Hispanic populations, significant disparities exist between SGMs and non-SGMs in victimization rates across all types of violent crime; in the Black population there are significant disparities between SGMs and non-SGMs in serious violent victimizations but not in simple assaults and violent victimizations that involve injuries.

Overall, there were notable differences in patterns of LGB-related differences in victimization rates at the intersection of sex and race. Among White respondents, the differences in violent victimization rates were significantly different by sex, with LGB White males and females reporting higher rates than their non-LGB counterparts. However, among Hispanic respondents, the LGB to non-LGB differences in victimization rates were only significant for females; among Black respondents, the rate differences were significant only for males. It is unclear what accounts for these differential rates of victimization at the intersection of sex, race and LGB identification. Regarding experiences with victimization among Black respondents, it could be that the overall higher rates of serious types of victimization among Black SGM people is driven by the experiences of Black LGB males. Similarly, it is possible that the overall high rates of victimization among SGM Hispanic respondents may be driven by higher rates among LGB females. For example, one study of sex and race differences in types of victimization showed that Black males had the highest levels of violent victimization and Hispanic females were highly likely to experience minor forms of victimization persistently across their lifespans [29]. Future research should examine qualitatively how SGM people across racialized groups describe the context and characteristics of experienced victimization to understand how sex plays a role in the types of victimization experienced.

For this study, we combined subgroups of SGM people (e.g., cisgender gay men, transgender men across sexual orientations) and collapsed potentially important subtypes of victimization by people who are well-known to the respondent (e.g., intimate partners and relatives) which may have masked important intersections of experiences, such as rates of intimate partner violence among Black transgender women compared to all other groups [see e.g. 30, 31]. For instance, Bender and Laurisen [4] find that bisexuals have disproportionate victimization experiences, and Truman and Morgan [8] show this pattern is consistent by race or ethnicity. Our limited subgroup analyses also demonstrated that bisexual women’s rates of victimization drove the observed rates among sexual minority females. Thus, finer-grained categories could reveal distinct victimization experiences along numerous axes of marginalization and characteristics of crime. Due to sample size limitations, we kept our analyses focused on broader categories.

There were racial differences in the relationship between SGM status and victimization characteristics. Namely, SGM Black respondents were the least likely to report victimization incidents to the police than their non-SGM counterparts. General population research has shown that Black women are more likely than White women to report intimate partner violence to the police despite existing highly strained relationships between Black communities and police departments, possibly as a function of the severity of violence being experienced [32]. It is reasonable to assume that these prior studies using NCVS data showing this paradoxical relationship to requesting police assistance among Black women was conducted with a majority cisgender heterosexual sample. As such, the difference we identified between SGM and non-SGM Black respondents in willingness to report the crime, and in particular the low rate of reporting incidents among Black SGM respondents, may be driven by the unique experiences of Black SGM people, such as the specific types of violence and expectations of police response. Our current analyses are limited by sample size to analyze more specific types of violence and police response. Future research with additional waves of data can provide the statistical power for such analyses.

### Conclusion

Our study contributes to understanding how multiple social status categories may result in unique victimization rates and experiences. To understand this, we examined victimization rates between sexual and gender identity groups within the three largest racial/ethnic categories in the U.S. Due to the quantitative nature of the data and that the surveys are not designed to assess subgroup specific factors relevant to victimization at related to combinations of gender, race/ethnic and SGM groups, we are limited in describing the intersectional pathways that lead to such experiences [e.g. as discussed in 11] and lack a more thorough understanding of the unique ways victimizations may affect people of various races or ethnicities, genders, and sexualities [e.g., see 33 for a discussion of methodological issues in establishing evidence of intersectionality]. Yet, the findings indicate a complex picture of how these social statuses interact that should be explored further.

## Supporting information

S1 Appendix Results for the other/unknown group. (DOCX)

S2 Appendix Adjusted victimization rates. (DOCX)
