# Supplementary material for: Violent victimization at the intersections of sexual orientation, gender identity, and race: National Crime Victimization Survey, 2017–2019
Source: PLoS One. 2023 Feb 9;18(2):e0281641. doi: 10.1371/journal.pone.0281641 (PMC9910698; doi:10.1371/journal.pone.0281641)
Supplement: S1 Appendix — (DOCX) [file pone.0281641.s001.docx]

**S1 Appendix. Results for the Other/Unknown Group.**

The main text of the article provides results for those who are SGM and non-SGM, but there are some respondents who could not be categories due to their responses to the sexual orientation, sex assigned at birth, and the current gender identity items. We categorize these individuals as “other/unknown.” Table S1.1 provides demographics of these respondents. Table S1.2 provides victimization rates for types of crime reported in the main text, and Table S1.3 provides violent victimization rates by sex. Table S1.4 provides victimization characteristics for respondents in this group.

**Table S1.1. Demographics of those in the other/unknown group.**

|  | White, non-Hispanic | | Black, non-Hispanic | | Hispanic or Latino | |
| --- | --- | --- | --- | --- | --- | --- |
|  | **%** | *SE* | % | *SE* | **%** | *SE* |
| **Sex** |  |  |  |  |  |  |
| Male | **46.6** | 0.70 | **41.9** | 1.88 | **50.2** | 1.61 |
| Female | **53.4** | 0.70 | **58.1** | 1.88 | **49.8** | 1.61 |
| *N* | 10,891 |  | 1,891 |  | 1,620 |  |
| **Age** |  |  |  |  |  |  |
| 16-17 | **2.8** | 0.28 | **2.6** | 0.54 | **4.3** | 0.84 |
| 18-24 | **9.8** | 0.62 | **9.8** | 1.1 | **13.5** | 1.45 |
| 25-34 | **14.4** | 0.62 | **20.2** | 1.26 | **21.9** | 1.33 |
| 35-49 | **19.7** | 0.68 | **25.9** | 1.38 | **28.6** | 1.65 |
| 50-64 | **28.3** | 0.79 | **25.5** | 1.33 | **20.9** | 1.61 |
| 65 or older | **25.1** | 0.88 | **16.1** | 1.23 | **10.8** | 1.04 |
| *N* | 10,891 |  | 1,891 |  | 1,620 |  |
| **Education** | |  |  |  |  |  |
| Less than High School | **11.6** | 0.56 | **16.4** | 1.43 | **31.0** | 1.94 |
| High School Graduate | **24.1** | 0.73 | **27.2** | 1.61 | **26.3** | 1.99 |
| Some College | **27.2** | 0.79 | **31.6** | 1.79 | **24.1** | 1.85 |
| Bachelor's Degree | **23.6** | 0.86 | **16.9** | 1.51 | **12.6** | 1.28 |
| Post-Graduate | **13.5** | 0.76 | **7.9** | 0.89 | **6.0** | 0.83 |
| *N* | 9,599 |  | 1,667 |  | 1,412 |  |
| **Marital Status** | |  |  |  |  |  |
| Never married | **36.5** | 1.06 | **52.9** | 2.25 | **46.3** | 1.76 |
| Married | **38.9** | 1.12 | **22.9** | 1.60 | **37.4** | 1.81 |
| Widowed | **8.6** | 0.52 | **6.6** | 0.88 | **3.7** | 0.66 |
| Divorced | **14.4** | 0.56 | **13.1** | 1.08 | **8.6** | 0.98 |
| Separated | **1.5** | 0.17 | **4.5** | 0.82 | **4.0** | 0.66 |
| *N* | 10,045 |  | 1,690 |  | 1,503 |  |
| **Household income** | |  |  |  |  |  |
| Less than $10,000 | **7.1** | 0.70 | **10.8** | 1.07 | **8.9** | 0.84 |
| $10,000-$14,999 | **4.5** | 0.38 | **8.1** | 1.06 | **5.4** | 0.65 |
| $15,000-$24,999 | **10.5** | 0.83 | **12.3** | 1.07 | **13.5** | 1.82 |
| $25,000-$34,999 | **10.5** | 0.66 | **12.1** | 1.11 | **12.8** | 1.13 |
| $35,000-$49,999 | **14.9** | 0.70 | **18.6** | 1.32 | **18.9** | 1.45 |
| $50,000-$74,999 | **19.0** | 0.77 | **17.2** | 1.50 | **17.3** | 1.61 |
| $75,000-$99,999 | **13.1** | 0.57 | **10.3** | 1.09 | **11.0** | 1.07 |
| $100,000 or more | **20.5** | 1.03 | **10.6** | 0.97 | **12.1** | 1.21 |
| *N* | 10,891 |  | 1,891 |  | 1,620 |  |
| **Urbanicity of Residence** | | |  |  |  |  |
| Urban | **32.3** | 1.97 | **55.6** | 2.37 | **51.2** | 2.54 |
| Suburban | **54.7** | 2.42 | **40.6** | 2.36 | **43.8** | 2.59 |
| Rural | **13.0** | 2.18 | **3.76** | 1.00 | **5.0** | 1.41 |
| *N* | 10,891 |  | 1,891 |  | 1,620 |  |

**Table S1.2. Victimization rates for the other/unknown group by race/ethnicity.**

|  | White, non-Hispanic | | Black, non-Hispanic | | Hispanic or Latino | |
| --- | --- | --- | --- | --- | --- | --- |
| Violent Crime | Rate per 1,000 | *SE* | Rate per 1,000 | *SE* | Rate per 1,000 | *SE* |
| Total | 25.3 | 3.8 | 23.2 | 5.4 | 79.7 | 24.9 |
| Simple Assault | 17.3 | 3.0 | 12.2 | 4.0 | 27.9 | 10.2 |
| Serious Violence | 8.0 | 1.8 | 11.0 | 3.4 | 51.8 | 24.2 |
| Involving Injury | 2.4 | 0.9 | 4.6^a^ | 1.8 | 32.5^a^ | 22.1 |
| Well-known | 6.0 | 1.6 | 9.6 | 3.9 | 49.4^a^ | 26.5 |
| Stranger | 13.2 | 2.9 | 5.4 | 1.8 | 21.1 | 8.5 |

^a^ Estimate is unreliable.

**Table S1.3. Violent victimization rates for the other/unknown group by sex and race/ethnicity.**

|  | White, Non-Hispanic | | | | | |
| --- | --- | --- | --- | --- | --- | --- |
|  | White, non-Hispanic | | Black, non-Hispanic | | Hispanic or Latino | |
| Violent Crime | Rate per 1,000 | *SE* | Rate per 1,000 | *SE* | Rate per 1,000 | *SE* |
| Males | 24.7 | 5.6 | 13.6 | 6.3 | 89.5 | 43.3 |
| Females | 25.8 | 5.1 | 30.2 | 8.0 | 69.8 | 24.8 |

**Table S1.4. Victimization characteristics for other/unknown by race or ethnicity.**

|  | White, non-Hispanic | | Black, non-Hispanic | | Hispanic or Latino | |
| --- | --- | --- | --- | --- | --- | --- |
|  | **%** | *SE* | % | *SE* | **%** | *SE* |
| **Reported to Police** | | | | | | |
| Yes | **35.4** | 9.77 | **40.0** | 8.77 | **27.2** | 10.9 |
| **Most Important Reason to Report (among incidents reported to the police)** | | | | | | |
| Get help with this incident | **11.2** | 5.07 | **29.0** | 13.24 | **60.2** | 17.34 |
| Recover loss | **0^a^** | -- | **1.3** | 1.33 | **0^a^** | -- |
| To get offender | **14.1** | 5.81 | **4.1** | 3.45 | **13.6** | 12.70 |
| Let police know | **0.9** | 0.59 | **5.1** | 5.03 | **3.3** | 3.44 |
| Other | **14.1** | 6.9 | **19.4** | 12.1 | **18.0** | 11.6 |
| **Most Important Reason to Not Report (among incidents not reported to the police)** | | | | | | |
| Dealt with another way | **26.4** | 10.62 | **55.5** | 15.22 | **45.1^a^** | 26.32 |
| Not important enough | **10.3** | 4.62 | **13.3** | 7.58 | **5.6** | 4.20 |
| Police couldn’t do anything | **0.6** | 0.43 | **0** | -- | **0** | -- |
| Police wouldn’t help | **9.4** | 4.49 | **19.7** | 11.91 | **19.1** | 15.60 |
| Other | **50.7^a^** | 17.62 | **11.5** | 9.71 | **14.2** | 9.09 |
| **Arrest Made (among incidents reported to the police)** | | | | | | |
| Yes | **27.7** | 10.9 | **27.9** | 13.2 | **7.9** | 6.0 |
| **Suspect Incident Was a Hate Crime** | | | | | | |
| Yes | **23.5** | 15.86 | **9.3** | 5.82 | **4.5** | 3.07 |
| **Seek Professional Help for Feelings Experienced as Victim of Crime** | | | | | | |
| Yes | **28.9** | 14.99 | **8.6** | 5.59 | **33.8^a^** | 21.25 |

^a^ Estimate is unreliable.
