# Supplementary material for: Violent victimization at the intersections of sexual orientation, gender identity, and race: National Crime Victimization Survey, 2017–2019
Source: PLoS One. 2023 Feb 9;18(2):e0281641. doi: 10.1371/journal.pone.0281641 (PMC9910698; doi:10.1371/journal.pone.0281641)
Supplement: S2 Appendix — (DOCX) [file pone.0281641.s002.docx]

**S2 Appendix. Adjusted Victimization Rates.**

The main text of the manuscript reports unadjusted victimization rates and compares those rates by SGM status and by sexuality and gender. Tables S2.1-S2.3 provide regression results on victimization rates for White, non-Hispanic, Black, non-Hispanic, and Hispanic or Latino samples, respectively. Each dependent variables have two sets of regressions with the first modeling SGM status on victimization rates and the second modeling the interaction of sexuality and gender. These models control for age cohort, educational attainment, marital status, household income, and urbanicity.

We use model predictions to provide adjusted victimization rates in Table S2.4 by SGM status and race or ethnicity. We also report test statistics and associated *p*-values examining the difference in victimization rates between SGMs and non-SGMs. Overall, disparities described in the main text remain after adjusting for demographic characteristics

We also use model predictions to provide adjusted violent victimization rates in Table S2.5 by sexuality, gender, and race or ethnicity. We similarly report test statistics and associated *p*-values examining the differences between LGB people and straight people. The adjusted rates show disparities similar to those reported in the main text.

**Table S2.1. Regression results on victimization rates non-Hispanic White respondents.**

|  | (1) | (2) | (3) | (4) | (5) | (6) | (7) |
| --- | --- | --- | --- | --- | --- | --- | --- |
| SGM | 61.2*** | 38.0* | 23.3*** | 11.8*** | 22.9*** | 33.6* |  |
|  | (17.9) | (16.9) | (3.98) | (3.38) | (5.47) | (16.0) |  |
| Other | 17.8^ | 16.5^ | 1.23 | -0.34 | 6.27 | 10.6* |  |
|  | (10.3) | (9.87) | (2.00) | (1.06) | (8.73) | (4.99) |  |
| Female | 3.27^ | 1.29 | 1.98** | 1.94*** | 6.36*** | -3.65** | 1.59 |
|  | (1.68) | (1.43) | (0.67) | (0.42) | (0.93) | (1.18) | (1.42) |
| LGB |  |  |  |  |  |  | 16.9* |
|  |  |  |  |  |  |  | (7.42) |
| LGB X Female |  |  |  |  |  |  | 83.3* |
|  |  |  |  |  |  |  | (34.2) |
| 18-24 | 2.71 | -2.35 | 5.06 | 1.65 | -1.59 | 6.50^ | 2.84 |
|  | (9.40) | (7.92) | (4.06) | (2.07) | (7.79) | (3.47) | (9.39) |
| 25-34 | 0.40 | 1.03 | -0.63 | -1.06 | -1.37 | 4.59 | 1.21 |
|  | (9.43) | (7.95) | (3.64) | (1.90) | (7.78) | (3.50) | (9.41) |
| 35-49 | -9.02 | -6.88 | -2.14 | -1.87 | -4.85 | -1.30 | -7.83 |
|  | (9.32) | (7.90) | (3.76) | (1.98) | (7.57) | (3.31) | (9.26) |
| 50-64 | -14.1 | -9.07 | -5.03 | -2.73 | -8.26 | -3.16 | -12.7 |
|  | (9.21) | (7.88) | (3.61) | (1.86) | (7.68) | (3.12) | (9.14) |
| 65+ | -25.9** | -17.5* | -8.39* | -4.08* | -12.3 | -6.68* | -24.5** |
|  | (9.09) | (7.78) | (3.55) | (1.81) | (7.50) | (3.13) | (9.03) |
| HS Grad | -3.89 | -2.67 | -1.22 | -0.31 | -1.97 | -1.37 | -3.93 |
|  | (3.28) | (2.29) | (2.09) | (0.92) | (2.09) | (1.47) | (3.27) |
| Some College | 0.57 | 1.92 | -1.34 | -0.16 | -1.90 | 2.99^ | 0.54 |
|  | (3.22) | (2.30) | (2.01) | (1.01) | (2.07) | (1.67) | (3.22) |
| BA | -4.06 | -1.83 | -2.23 | -0.59 | -3.81^ | 1.31 | -3.89 |
|  | (3.46) | (2.32) | (2.10) | (1.00) | (1.94) | (1.86) | (3.47) |
| Post-Grad | -2.08 | 0.72 | -2.80 | -0.61 | -2.11 | -1.15 | -1.94 |
|  | (3.77) | (2.89) | (2.06) | (1.08) | (2.16) | (1.61) | (3.77) |
| Widowed | 0.86 | 0.35 | 0.51 | -0.50 | -0.084 | 0.13 | 1.42 |
|  | (1.96) | (1.46) | (0.93) | (0.32) | (1.21) | (0.93) | (1.92) |
| Divorced | 17.2*** | 9.96*** | 7.21*** | 2.96*** | 10.1*** | 4.17** | 17.4*** |
|  | (2.24) | (1.86) | (1.30) | (0.77) | (1.53) | (1.28) | (2.25) |
| Separated | 59.0*** | 45.4*** | 13.6** | 5.30* | 35.6*** | 10.6^ | 59.0*** |
|  | (10.7) | (9.46) | (4.75) | (2.54) | (7.25) | (6.37) | (10.7) |
| Never married | 5.98* | 4.35^ | 1.63 | 0.33 | 2.90^ | 0.81 | 7.06** |
|  | (2.61) | (2.38) | (1.13) | (0.59) | (1.48) | (1.68) | (2.67) |
| Residue | -11.4^ | -6.47 | -4.97*** | -1.13 | -2.91 | -5.32 | -7.09 |
|  | (6.05) | (5.88) | (0.95) | (0.77) | (3.38) | (4.24) | (5.67) |
| 10K-15K | -8.21 | 2.86 | -11.1* | -7.64** | -0.093 | -3.00 | -7.94 |
|  | (10.6) | (9.72) | (4.31) | (2.53) | (7.52) | (7.26) | (10.5) |
| 15K-25K | -24.4** | -11.1 | -13.2*** | -7.87** | -7.19^ | -10.4 | -24.1** |
|  | (8.02) | (6.83) | (3.87) | (2.41) | (4.02) | (6.79) | (7.96) |
| 25K-35K | -24.4** | -10.3 | -14.1*** | -8.66*** | -8.53* | -7.40 | -24.2** |
|  | (7.81) | (6.90) | (3.75) | (2.46) | (3.82) | (6.91) | (7.74) |
| 35K-50K | -27.9*** | -13.0^ | -14.9*** | -9.19*** | -11.7*** | -9.27 | -27.8*** |
|  | (8.09) | (7.17) | (3.65) | (2.37) | (3.48) | (7.19) | (8.02) |
| 50K-75K | -30.1*** | -13.5^ | -16.6*** | -9.13*** | -11.6** | -9.88 | -30.0*** |
|  | (7.89) | (6.90) | (3.66) | (2.46) | (3.65) | (6.97) | (7.79) |
| 75K-100K | -34.3*** | -15.4* | -18.9*** | -10.8*** | -14.1*** | -10.8 | -34.3*** |
|  | (7.61) | (6.82) | (3.60) | (2.39) | (3.50) | (6.88) | (7.51) |
| 100K+ | -33.9*** | -15.6* | -18.2*** | -10.4*** | -13.5*** | -10.7 | -33.8*** |
|  | (7.60) | (6.83) | (3.63) | (2.39) | (3.51) | (6.88) | (7.51) |
| (S)MSA not city | -7.75*** | -5.12** | -2.64** | 0.26 | 2.64** | -7.37*** | -8.11*** |
|  | (2.10) | (1.61) | (0.93) | (0.50) | (1.01) | (1.29) | (2.09) |
| Not (S)MSA | -8.41* | -5.54* | -2.87* | -0.42 | 4.32* | -10.6*** | -8.82** |
|  | (3.30) | (2.55) | (1.35) | (0.77) | (2.11) | (1.49) | (3.26) |
| Constant | 62.2*** | 35.2** | 27.0*** | 12.4*** | 18.9* | 25.0** | 62.3*** |
|  | (11.9) | (10.6) | (4.89) | (2.75) | (8.12) | (7.79) | (11.9) |
| Observations | 462101 | 462101 | 462101 | 462101 | 462101 | 462101 | 462101 |
| R-squared | 0.0025 | 0.0015 | 0.0016 | 0.00092 | 0.0015 | 0.0014 | 0.0027 |
| F | 19.6 | 11.3 | 10.9 | 4.71 | 9.13 | 10.1 | 18.9 |
| df_m | 26 | 26 | 26 | 26 | 26 | 26 | 26 |
| df_r | 170 | 170 | 170 | 170 | 170 | 170 | 170 |

Dependent variables are (1) violent victimizations; (2) simple assaults; (3) serious violence; (4) serious violence involving injury; (5) well-known victimizations; (6) stranger victimizations; (7) violent victimizations. df_m = numerator degrees of freedom; df_r = denominator degrees of freedom.

^ *p* < .10; * *p* < .05; ** *p* < .01; *** *p* < .001 (two-tailed).

**Table S2.2. Regression results on victimization rates non-Hispanic Black respondents.**

|  | (1) | (2) | (3) | (4) | (5) | (6) | (7) |
| --- | --- | --- | --- | --- | --- | --- | --- |
| SGM | 36.9^ | 17.3 | 19.6^ | 6.98 | 14.6 | 15.4 |  |
|  | (19.2) | (11.3) | (10.8) | (4.88) | (8.85) | (11.7) |  |
| Other | 19.4* | 8.27^ | 11.1 | 1.20 | 13.3^ | 0.78 |  |
|  | (8.48) | (4.88) | (6.81) | (1.51) | (6.93) | (2.90) |  |
| Female | -5.29 | -1.01 | -4.29* | -0.11 | 4.12** | -4.32** | -5.00 |
|  | (3.23) | (2.14) | (2.10) | (0.88) | (1.29) | (1.41) | (3.17) |
| LGB |  |  |  |  |  |  | 44.5^ |
|  |  |  |  |  |  |  | (26.5) |
| LGB X Female |  |  |  |  |  |  | -14.4 |
|  |  |  |  |  |  |  | (37.0) |
| 18-24 | 9.65 | 2.77 | 6.88 | 3.88* | 7.89 | 0.11 | 9.72 |
|  | (9.96) | (8.01) | (5.96) | (1.87) | (4.94) | (5.92) | (9.93) |
| 25-34 | 1.06 | -3.37 | 4.43 | 3.68 | 5.15 | -4.33 | 1.21 |
|  | (9.60) | (7.94) | (6.03) | (2.39) | (4.55) | (5.42) | (9.58) |
| 35-49 | 8.24 | 1.21 | 7.03 | 4.04^ | 5.18 | -1.02 | 8.47 |
|  | (10.4) | (8.86) | (6.24) | (2.30) | (4.57) | (5.65) | (10.4) |
| 50-64 | -1.92 | -4.99 | 3.08 | 1.55 | 0.43 | -6.57 | -1.65 |
|  | (10.5) | (8.43) | (6.66) | (1.83) | (4.55) | (5.74) | (10.4) |
| 65+ | -15.9 | -9.64 | -6.26 | 0.89 | -2.02 | -13.2* | -15.5 |
|  | (11.0) | (8.94) | (6.76) | (1.80) | (4.78) | (6.52) | (10.9) |
| HS Grad | -4.70 | 1.79 | -6.49^ | -1.27 | -3.92^ | 1.43 | -4.74 |
|  | (5.36) | (2.97) | (3.86) | (1.50) | (2.32) | (2.23) | (5.35) |
| Some College | 2.46 | 2.69 | -0.23 | -0.89 | -0.0045 | 4.62^ | 2.49 |
|  | (5.34) | (2.72) | (4.06) | (1.41) | (2.62) | (2.66) | (5.33) |
| BA | -5.40 | 2.31 | -7.71* | -3.28* | -5.20* | 0.58 | -5.32 |
|  | (5.43) | (3.74) | (3.67) | (1.43) | (2.33) | (2.05) | (5.43) |
| Post-Grad | -4.34 | 1.46 | -5.80 | -1.43 | -1.30 | 0.030 | -4.34 |
|  | (5.69) | (3.47) | (3.99) | (2.22) | (3.19) | (2.24) | (5.68) |
| Widowed | 17.5 | 5.91^ | 11.6 | -0.23 | 3.53 | 14.7 | 17.5 |
|  | (10.8) | (3.28) | (10.2) | (1.04) | (3.01) | (10.2) | (10.8) |
| Divorced | 12.6** | 10.9** | 1.70 | 0.50 | 2.33 | 7.12* | 12.8** |
|  | (4.49) | (3.80) | (1.95) | (1.12) | (1.88) | (3.17) | (4.50) |
| Separated | 16.4* | 11.5^ | 4.93 | 1.94 | 8.29^ | 2.86 | 16.7* |
|  | (7.62) | (6.55) | (3.67) | (2.08) | (4.57) | (2.73) | (7.62) |
| Never married | 4.04 | 1.88 | 2.16 | -0.033 | -0.034 | 1.55 | 4.34 |
|  | (2.93) | (2.31) | (1.97) | (1.00) | (1.58) | (1.24) | (2.92) |
| Residue | -1.63 | 0.57 | -2.20 | -2.91** | -1.52 | 6.17 | 1.05 |
|  | (11.8) | (10.7) | (5.89) | (0.88) | (5.81) | (10.7) | (11.6) |
| 10K-15K | -24.7** | -9.41^ | -15.3* | -2.40 | -6.16 | -7.04 | -24.7** |
|  | (9.40) | (5.40) | (6.76) | (2.88) | (5.34) | (4.38) | (9.40) |
| 15K-25K | -32.0*** | -13.8** | -18.2** | -3.53* | -12.3*** | -10.1* | -32.0*** |
|  | (8.35) | (4.46) | (5.79) | (1.62) | (3.59) | (4.23) | (8.35) |
| 25K-35K | -40.0*** | -20.3*** | -19.6** | -6.02*** | -15.5*** | -12.5** | -40.0*** |
|  | (8.54) | (4.24) | (6.02) | (1.38) | (3.93) | (4.15) | (8.55) |
| 35K-50K | -42.1*** | -18.5*** | -23.6*** | -5.75*** | -14.1*** | -13.0** | -42.1*** |
|  | (8.07) | (4.14) | (5.85) | (1.48) | (3.79) | (4.15) | (8.08) |
| 50K-75K | -37.7*** | -14.0** | -23.7*** | -6.40*** | -17.1*** | -8.27^ | -37.7*** |
|  | (8.22) | (4.61) | (5.77) | (1.50) | (3.53) | (4.46) | (8.23) |
| 75K-100K | -40.8*** | -19.3*** | -21.5** | -6.63*** | -18.2*** | -8.96 | -40.8*** |
|  | (9.21) | (4.38) | (7.33) | (1.44) | (3.53) | (6.11) | (9.20) |
| 100K+ | -39.5*** | -19.2*** | -20.3*** | -2.39 | -14.1*** | -13.2*** | -39.6*** |
|  | (7.85) | (4.30) | (5.68) | (2.37) | (3.80) | (3.82) | (7.86) |
| (S)MSA not city | -9.37** | -5.12* | -4.25* | -0.73 | -1.05 | -4.92** | -9.43** |
|  | (3.07) | (2.03) | (2.00) | (0.79) | (1.40) | (1.85) | (3.07) |
| Not (S)MSA | -13.8** | -8.01** | -5.77* | -0.059 | -2.32 | -5.87* | -14.1** |
|  | (4.39) | (2.62) | (2.89) | (1.17) | (2.60) | (2.39) | (4.41) |
| Constant | 58.8*** | 28.1** | 30.8*** | 6.14** | 15.9** | 22.4** | 58.9*** |
|  | (12.8) | (9.91) | (7.51) | (1.98) | (5.12) | (6.83) | (12.8) |
| Observations | 72730 | 72730 | 72730 | 72730 | 72730 | 72730 | 72730 |
| R-squared | 0.0031 | 0.0017 | 0.0022 | 0.00087 | 0.0022 | 0.0016 | 0.0030 |
| F | 3.24 | 2.43 | 2.57 | 3.49 | 3.22 | 2.58 | 3.23 |
| df_m | 26 | 26 | 26 | 26 | 26 | 26 | 26 |
| df_r | 169 | 169 | 169 | 169 | 169 | 169 | 169 |

Dependent variables are (1) violent victimizations; (2) simple assaults; (3) serious violence; (4) serious violence involving injury; (5) well-known victimizations; (6) stranger victimizations; (7) violent victimizations. df_m = numerator degrees of freedom; df_r = denominator degrees of freedom.

^ *p* < .10; * *p* < .05; ** *p* < .01; *** *p* < .001 (two-tailed).

**Table S2.3. Regression results on victimization rates Hispanic respondents.**

|  | (1) | (2) | (3) | (4) | (5) | (6) | (7) |
| --- | --- | --- | --- | --- | --- | --- | --- |
| SGM | 44.2* | 19.9* | 24.2 | 6.98 | 16.6 | 17.2^ |  |
|  | (18.8) | (9.96) | (14.8) | (4.54) | (10.3) | (8.87) |  |
| Other | 61.4* | 20.2^ | 41.2^ | 33.7 | 47.2^ | 10.1 |  |
|  | (25.3) | (10.3) | (24.4) | (25.1) | (27.0) | (8.34) |  |
| Female | -0.45 | 0.023 | -0.47 | -0.11 | 4.48** | -4.09** | -1.54 |
|  | (2.24) | (1.59) | (1.73) | (1.11) | (1.48) | (1.24) | (2.22) |
| LGB |  |  |  |  |  |  | 16.2 |
|  |  |  |  |  |  |  | (15.0) |
| LGB X Female |  |  |  |  |  |  | 57.7 |
|  |  |  |  |  |  |  | (37.1) |
| 18-24 | 1.42 | 2.05 | -0.62 | -8.91 | -2.28 | 2.27 | 1.48 |
|  | (11.8) | (3.54) | (11.3) | (10.3) | (10.9) | (4.06) | (12.0) |
| 25-34 | 1.30 | 5.53^ | -4.23 | -9.02 | -3.40 | 3.43 | 2.14 |
|  | (11.4) | (3.32) | (10.7) | (10.0) | (10.2) | (3.94) | (11.4) |
| 35-49 | -3.06 | 2.29 | -5.35 | -8.51 | -6.23 | 0.91 | -1.83 |
|  | (11.7) | (3.90) | (11.3) | (10.4) | (10.6) | (4.26) | (11.7) |
| 50-64 | -5.99 | 4.25 | -10.2 | -11.6 | -8.39 | -0.26 | -4.39 |
|  | (12.2) | (4.49) | (11.5) | (10.5) | (10.8) | (4.41) | (12.1) |
| 65+ | -17.9 | -4.24 | -13.7 | -12.2 | -12.8 | -4.40 | -16.5 |
|  | (11.5) | (4.15) | (11.2) | (10.3) | (10.5) | (4.17) | (11.5) |
| HS Grad | -1.16 | -0.85 | -0.30 | -1.14 | -3.83 | -0.50 | -1.17 |
|  | (3.21) | (2.22) | (2.27) | (1.18) | (2.33) | (1.84) | (3.22) |
| Some College | 4.77 | 4.77^ | 0.0012 | -0.37 | -1.72 | 2.83 | 4.80 |
|  | (3.70) | (2.67) | (2.57) | (1.31) | (2.70) | (2.45) | (3.72) |
| BA | -0.66 | 1.65 | -2.31 | -1.29 | -0.63 | -1.44 | -0.12 |
|  | (3.93) | (2.86) | (2.52) | (1.73) | (2.74) | (2.45) | (3.93) |
| Post-Grad | 4.55 | 6.89 | -2.34 | -2.75 | -1.60 | 1.24 | 5.12 |
|  | (5.85) | (4.36) | (3.58) | (2.11) | (3.47) | (2.87) | (5.80) |
| Widowed | 9.81 | 8.90 | 0.91 | -0.38 | 0.16 | 7.71 | 10.7 |
|  | (6.70) | (6.58) | (1.94) | (0.61) | (1.67) | (6.20) | (6.62) |
| Divorced | 21.1*** | 11.4** | 9.69** | 4.62^ | 12.7** | 6.59* | 21.6*** |
|  | (5.42) | (4.19) | (3.52) | (2.54) | (3.84) | (3.03) | (5.47) |
| Separated | 21.5** | 9.53^ | 12.0* | 10.5* | 11.7* | 3.58 | 22.2** |
|  | (8.05) | (5.12) | (5.13) | (4.57) | (5.33) | (3.46) | (8.02) |
| Never married | 11.0** | 3.96 | 7.00*** | 1.82^ | 2.21 | 3.81 | 12.3*** |
|  | (3.52) | (2.56) | (2.06) | (0.96) | (1.97) | (2.34) | (3.48) |
| Residue | -20.1*** | -9.91*** | -10.2** | -4.82^ | -8.51* | -7.70*** | -13.9*** |
|  | (3.99) | (2.00) | (3.09) | (2.66) | (3.34) | (1.31) | (1.84) |
| 10K-15K | -21.9* | -4.31 | -17.6^ | -7.97^ | -14.9^ | -3.23 | -22.8* |
|  | (10.7) | (4.65) | (9.19) | (4.44) | (8.38) | (2.39) | (10.8) |
| 15K-25K | -23.6* | -2.00 | -21.6* | -10.7* | -17.4* | 2.73 | -24.2* |
|  | (10.6) | (5.05) | (9.18) | (4.30) | (7.65) | (3.42) | (10.6) |
| 25K-35K | -21.6* | -0.83 | -20.8* | -11.2** | -17.4* | 1.11 | -22.5* |
|  | (10.7) | (4.80) | (9.21) | (3.94) | (7.54) | (3.33) | (10.8) |
| 35K-50K | -28.0** | -4.00 | -24.0** | -10.8** | -19.5** | -0.67 | -28.7** |
|  | (9.61) | (4.01) | (8.81) | (3.91) | (7.04) | (2.45) | (9.63) |
| 50K-75K | -30.4** | -6.08 | -24.3** | -12.2** | -20.7** | -1.25 | -31.1** |
|  | (9.55) | (4.09) | (8.69) | (3.90) | (7.16) | (2.37) | (9.56) |
| 75K-100K | -30.8** | -3.80 | -27.0** | -12.7*** | -20.6** | 0.70 | -31.5** |
|  | (9.75) | (4.84) | (8.75) | (3.71) | (7.04) | (2.98) | (9.78) |
| 100K+ | -26.3* | -5.52 | -20.8* | -7.10 | -16.3* | 1.04 | -27.0* |
|  | (10.6) | (4.48) | (9.28) | (5.30) | (7.67) | (3.11) | (10.5) |
| (S)MSA not city | -5.12* | -3.62* | -1.51 | 0.58 | 0.21 | -3.50* | -5.41* |
|  | (2.51) | (1.71) | (1.73) | (1.00) | (1.56) | (1.67) | (2.48) |
| Not (S)MSA | 2.63 | 1.15 | 1.48 | 5.35 | 6.20 | -5.68* | 2.37 |
|  | (5.55) | (3.97) | (4.29) | (4.04) | (5.34) | (2.62) | (5.49) |
| Constant | 42.6** | 8.32 | 34.3* | 20.8^ | 25.7* | 8.83^ | 43.5** |
|  | (15.0) | (5.49) | (13.8) | (10.8) | (12.8) | (4.48) | (15.3) |
| Observations | 81908 | 81908 | 81908 | 81908 | 81908 | 81908 | 81908 |
| R-squared | 0.0028 | 0.0010 | 0.0027 | 0.0024 | 0.0021 | 0.0011 | 0.0023 |
| F | 5.67 | 3.22 | 8.45 | 2.73 | 3.50 | 6.28 | 9.25 |
| df_m | 26 | 26 | 26 | 26 | 26 | 26 | 26 |
| df_r | 170 | 170 | 170 | 170 | 170 | 170 | 170 |

Dependent variables are (1) violent victimizations; (2) simple assaults; (3) serious violence; (4) serious violence involving injury; (5) well-known victimizations; (6) stranger victimizations; (7) violent victimizations. df_m = numerator degrees of freedom; df_r = denominator degrees of freedom.

^ *p* < .10; * *p* < .05; ** *p* < .01; *** *p* < .001 (two-tailed).

**Table S2.4. Adjusted victimization rates.**

|  | White, Non-Hispanic | | | | | | Black, Non-Hispanic | | | | | | Hispanic | | | | | |
| --- | --- | --- | --- | --- | --- | --- | --- | --- | --- | --- | --- | --- | --- | --- | --- | --- | --- | --- |
|  | SGM | | Non-SGM | | Statistics | | SGM | | Non-SGM | | Statistics | | SGM | | Non-SGM | | Statistics | |
| Violent Crime | Rate per 1,000 | *SE* | Rate per 1,000 | *SE* | \|*t*\| | *p* | Rate per 1,000 | SE | Rate per 1,000 | SE | \|t\| | p | Rate per 1,000 | *SE* | Rate per 1,000 | *SE* | \|*t*\| | *p* |
| Total | 81.8 | 17.8 | 20.5 | 0.8 | 3.42 | .001 | 57.6 | 19.4 | 20.7 | 1.5 | 1.92 | .056 | 63.3 | 18.8 | 19.2 | 1.1 | 2.34 | .020 |
| Simple Assault | 51.7 | 16.8 | 13.7 | 0.6 | 2.25 | .026 | 28.9 | 11.3 | 11.7 | 0.9 | 1.53 | .127 | 29.9 | 10.0 | 9.9 | 0.8 | 2.00 | .047 |
| Serious Violence | 30.0 | 3.9 | 6.8 | 0.4 | 5.85 | <.001 | 28.7 | 10.8 | 9.1 | 1.0 | 1.82 | .071 | 33.5 | 14.6 | 9.2 | 0.8 | 1.64 | .103 |
| Involving Injury | 14.1 | 3.3 | 2.3 | 0.2 | 3.48 | .001 | 9.8^a^ | 4.9 | 2.8 | 0.4 | 1.43 | .154 | 9.6 | 4.5 | 2.6 | 0.4 | 1.54 | .126 |
| Well-known | 30.0 | 5.4 | 7.1 | 0.4 | 4.18 | <.001 | 20.9 | 8.8 | 6.3 | 0.7 | 1.65 | .100 | 22.2 | 10.3 | 5.6 | 0.7 | 1.61 | .110 |
| Stranger | 41.6 | 16.0 | 8.0 | 0.5 | 2.10 | .037 | 23.1 | 11.7 | 7.7 | 0.9 | 1.31 | .191 | 25.8 | 8.8 | 8.5 | 0.7 | 1.94 | .054 |

^a^ Estimate is unreliable.

**Table S2.5. Adjusted victimization rates, by gender.**

|  | Men | | | | | | | | | | | | | | | | | |
| --- | --- | --- | --- | --- | --- | --- | --- | --- | --- | --- | --- | --- | --- | --- | --- | --- | --- | --- |
|  | White, Non-Hispanic | | | | | | Black, Non-Hispanic | | | | | | Hispanic | | | | | |
|  | GB | | Straight | | Statistics | | GB | | Straight | | Statistics | | GB | | Straight | | Statistics | |
| Violent Crime | Rate per 1,000 | *SE* | Rate per 1,000 | *SE* | \|*t*\| | *p* | Rate per 1,000 | SE | Rate per 1,000 | SE | \|*t*\| | *p* | Rate per 1,000 | *SE* | Rate per 1,000 | *SE* | \|*t*\| | *p* |
| Total | 37.1 | 7.3 | 20.2 | 1.2 | 2.28 | .024 | 68.5 | 26.8 | 24.1 | 2.6 | 1.14 | .258 | 37.4 | 15.0 | 21.2 | 1.8 | 1.08 | .282 |
|  | Women | | | | | | | | | | | | | | | | | |
|  | White, Non-Hispanic | | | | | | Black, Non-Hispanic | | | | | | Hispanic | | | | | |
|  | LB | | Straight | | Statistics | | LB | | Straight | | Statistics | | LB | | Straight | | Statistics | |
| Violent Crime |  |  |  |  |  |  |  |  |  |  |  |  |  |  |  |  |  |  |
| Total | 122.0 | 33.8 | 21.8 | 1.0 | 2.97 | .003 | 49.2 | 26.5 | 19.1 | 1.7 | 1.68 | .095 | 93.6 | 35.3 | 19.6 | 1.6 | 2.09 | .038 |
